# Analysis of tumor necrosis factor α-induced and nuclear factor κB-silenced LNCaP prostate cancer cells by RT-qPCR

**DOI:** 10.3892/etm.2014.2032

**Published:** 2014-10-17

**Authors:** CEREN GONEN-KORKMAZ, GULNUR SEVIN, GOKSEL GOKCE, MEHMET ZUHURI ARUN, GOKCE YILDIRIM, BUKET REEL, AYSEGUL KAYMAK, DENIZ OGUT

**Affiliations:** Department of Pharmacology, Faculty of Pharmacy, Ege University, Izmir 35100, Turkey

**Keywords:** prostate cancer, cell culture, gene silencing, NFκB, TNFα, p53, STAMP genes

## Abstract

Prostate cancer is the second leading cause of morbidity and mortality in males in the Western world. In the present study, LNCaP, which is an androgen receptor-positive and androgen-responsive prostate cancer cell line derived from lymph node metastasis, and DU145, which is an androgen receptor-negative prostate cancer cell line derived from brain metastasis, were investigated. TNFα treatment decreased p105 and p50 expression and R1881 treatment slightly decreased p105 expression but increased p50 expression with or without TNFα induction. As an aggressive prostate cancer cell line, DU145 transfected with six transmembrane protein of prostate (STAMP)1 or STAMP2 was also exposed to TNFα. Western blotting indicated that transfection with either STAMP gene caused a significant increase in NFκB expression following TNFα induction. In addition, following the treatment of LNCaP cells with TNFα, reverse transcription quantitative polymerase chain reaction (RT-qPCR) was performed with a panel of apoptosis-related gene primers. The apoptosis-related genes p53, p73, caspase 7 and caspase 9 showed statistically significant increases in expression levels while the expression levels of MDM2 and STAMP1 decreased following TNFα induction. Furthermore, LNCaP cells were transfected with a small interfering NFκB (siNFκB) construct for 1 and 4 days and induced with TNFα for the final 24 h. RT-qPCR amplifications were performed with apoptosis-related gene primers, including p53, caspases and STAMPs. However, no changes in the level of STAMP2 were observed between cells in the presence or absence of TNFα induction or between those transfected or not transfected with siNFκB; however, the level of STAMP1 was significantly decreased by TNFα induction, and significantly increased with siNFκB transfection. Silencing of the survival gene NFκB caused anti-apoptotic STAMP1 expression to increase, which repressed p53, together with MDM2. NFκB silencing had varying effects on a panel of cancer regulatory genes. Therefore, the effective inhibition of NFκB may be critical in providing a targeted pathway for prostate cancer prevention.

## Introduction

Prostate cancer is the most commonly diagnosed cancer and the second leading cause of cancer mortality in males in the Western world. Human prostate adenocarcinoma cell lines are normally resistant to programmed cell death, known as apoptosis (1).

Six transmembrane epithelial antigen of the prostate (STEAP) (2) belongs to the six transmembrane protein of prostate (STAMP) gene family, and is the first characterized transmembrane gene that is enriched in the prostate. STEAP is expressed in metastatic prostate cancer samples; in particular, STAMP1/STEAP2 (3) and STAMP2/STEAP4 (4) are expressed in the androgen receptor-positive prostate cancer cell line LNCaP, and androgen receptor-mediated regulation of STAMP2 has previously been demonstrated (4). The role of STAMP2 in metabolic disease and its function in the prevention of excessive inflammation and protection of adipocyte insulin sensitivity and systemic glucose homeostasis has been reported in mice (5). Other members of the STAMP family include pHyde, a rat protein that has been implicated in the apoptosis of prostate cancer cells (6), and its human homolog, tumor suppressor-activated pathway 6 (TSAP6), also known as STEAP3, a p53-inducible gene, involved in apoptosis and the cell cycle in prostate cancer and HeLa cells (7).

It is hypothesized that STAMP/STEAP family genes may have similar functions, with roles in the normal biology and pathophysiology of prostate cancer. Activation of extracellular signal-regulated kinase (ERK), which has previously been implicated in prostate cancer progression, was reported with ectopic expression of STAMP1 in DU145 cells and, conversely, was strongly downregulated in LNCaP cells following STAMP1 knockdown (8). The promoter regions of STAMP genes have been analyzed, and tumor suppressor gene p53 response elements and nuclear factor κB (NFκB) response elements identified and confirmed in the promoter region of STAMP genes (Gonen-Korkmaz *et al*, unpublished data). In the present study, tumor necrosis factor α (TNFα)-induced apoptosis in the LNCaP (human prostate adenocarcinoma lymph node metastasis) cell line was investigated by amplifications conducted using a panel of apoptosis-related gene primers. The LNCaP cell line expresses STAMP1 and STAMP2. Another prostate cancer cell line, DU145, which is derived from brain metastasis, was transfected with STAMP1 and STAMP2 and then induced by TNFα. The apoptosis/survival equilibrium, which is determined by NFκB, was investigated by western blot analysis of the two cell lines.

## Materials and methods

### Cell culture

LNCaP cells were cultured in RPMI-1640 (Gibco-BRL, Gaithersburg, MD, USA) with 10% fetal bovine serum (FBS), while DU145 cells were cultured in Dulbecco’s modified Eagle’s medium (DMEM)-Ham’s F12 (Gibco-BRL) with 5% FBS, 1% L-glutamine and 1 U/ml each of penicillin/streptomycin. Cells were incubated at 37°C with 5% CO_2_ in a humidified atmosphere. The cell lines were purchased from ATCC (Manassas, VA, USA).

### Primer design, plasmid construction and transfection

The full-length open reading frames of STAMP1 and STAMP2 were amplified using primers (10 pmol of each), designed using Light Cycler Probe Design Software 2 (Roche Diagnostics, Mannheim, Germany). The PCR product was cloned into pcDNA4-HisMax-TOPO (Invitrogen Life Technologies, Carlsbad, CA, USA) vector, in accordance with the manufacturer’s instructions. The inserts were verified by PCR amplifications. All transfections including small interfering RNA (siRNA) were performed using FuGENE HD (Roche Diagnostics) transfection reagent, in accordance with the manufacturer’s instructions. Briefly, cells were seeded in 6-well plates one day prior to transfection. The following day, the transfection solution was prepared in a 1.5-ml tube with 100 μl pre-warmed RPMI-1640 (without antibiotics), 1 μg pcDNA4-HisMax-gene plasmid DNA was added and the solution was incubated for 5 min. A total of 3 μl FuGENE HD transfection reagent was added dropwise with tapping to mix, and, following a 15-min incubation at room temperature, the transfection mix was added to the cells dropwise.

### siRNA-mediated knockdown of NFκB

LNCaP cells were transfected with either scrambled control siRNA (sc-37007) or NFκB-specific siRNA (sc-29410), purchased from Santa Cruz Biotechnology Inc. (Dallas, TX, USA). The sequences were provided by the manufacturer.

A total of 100 pmol siRNA (final concentration, 50 nM) was used to transfect cells with the aid of 10 μl FuGENE HD transfection reagent and the cells were incubated with the siRNA construct for 1 and 4 days, respectively, in accordance with the manufacturer’s instructions.

### Treatment of the cells

The LNCaP cells were divided into four groups. The control group was cultured in the absence of treatment for 24 h; the TNFα induction group was induced by TNFα (100 ng/ml; Sigma, St. Louis, MO, USA) for 24 h. the R1881 group was treated for 24 h with a synthetic androgen, R1881 (1×10^−8^ M; Sigma); and the TNF + R1881 group was treated concomitantly with TNFα (100 ng/ml) and R1881 (1×10^−8^ M) for 24 h.

DU145 cells transfected with STAMP1 or STAMP2 were induced by TNFα (100 ng/ml) or were not induced for 24 h.

In another series of experiments, following NFκB gene silencing, the LNCaP cells transfected for 1 day were induced by TNFα (100 ng/ml) or were not induced for a further 24 h. The cells transfected for 4 days were induced by TNFα (100 ng/ml) or were not induced at the third day of transfection.

### Reverse transcription quantitative polymerase chain reaction (RT-qPCR) using a panel of apoptosis-related gene primers

qPCR was performed using a Light Cycler^®^ 480 (Roche Diagnostics) instrument and Light Cycler 480 SYBR Green 1 Master kit (Roche Diagnostics). Briefly, the reactions were performed in a 20-μl volume with 5 pmol of each primer and 1 μl of cDNA template derived from reverse-transcribed RNA of scrambled siRNA (control) and NFκB siRNA-transfected cells. The primers used are shown in Table I. GAPDH, a human housekeeping gene, was used as an endogenous control and reference gene for relative quantifications. The same thermal profile was optimized for all primers: pre-incubation for 5 min at 95°C for 1 cycle, followed by 40 cycles of denaturation at 95°C for 10 sec, primer annealing at 64°C for 20 sec, and primer extension at 72°C for 10 sec. Water was included as a no-template control. Melting curves were derived after 40 cycles by a denaturation step at 95°C for 10 sec, followed by annealing at 65°C for 15 sec, and a temperature rise to 95°C with a heating rate of 0.1°C/sec and continuous fluorescence measurement. Final cooling was performed at 37°C for 30 sec. Melting curve analyses of each sample were performed using LightCycler 480 Software version LCS480 (Roche Diagnostics). The analysis step of relative quantification was a fully automated process accomplished by the software, with the efficiency set at 2 and the cDNA of untreated cells defined as the calibrator.

### Cell lysis, protein extraction and western blot analysis

For protein extraction, cells were grown on 60-mm culture dishes (Orange Scientific, Braine-l’Alleud, Belgium) and washed once with phosphate-buffered saline (PBS) prior to cell lysis. Cells were resuspended in 250 μl modified radioimmunoprecipitation assay (RIPA) lysis buffer (10 mM Tris Cl, pH 8.0; 1% Triton X-100; 0.1% SDS; 0.1% Na deoxycholate; 1 mM EDTA; 1 mM EGTA; 140 mM NaCl) containing protease and phosphatase inhibitors. Cells were collected from culture plates using a cell scraper and were transferred to Eppendorf tubes. Cells were incubated on ice for 1 h (with pipetting up/down every 10 min), centrifuged at 14,000 × g for 30 min and the cleared supernatants were then collected. The protein concentration was determined using the Qubit Protein assay kit (Invitrogen Life Technologies) where appropriate. SDS-PAGE and western blot analysis was performed under standard conditions using 20 μg lysate per lane. Proteins were separated on a 10% gel and transferred to a polyvinylidene difluoride (PVDF) membrane (Amersham Pharmacia Biotech, Amersham, UK) using a semi-dry transfer blotter (VWR International Ltd., Lutterworth, UK). The PVDF membrane was blocked with 10% dry milk in PBS solution containing 0.1% Tween 20 (PBS-T) for 10 min. Primary and secondary antibody incubations were performed using PBS-T containing 0.5% dry milk at 4°C overnight. Membranes were developed using enhanced chemiluminescence (ECL) plus reagent (Amersham Pharmacia Biotech) for 5 min, and images were captured using a FX7 dark room chemiluminescence camera (Vilber Lourmat, Marne-la-Vallée, France). The antibodies used were mouse anti-human NFκB (p50/p105) monoclonal antibody (sc-166588; Santa Cruz Biotechnology) used at a dilution of 1:1,000 and mouse anti-human β-actin monoclonal antibody (A5316; Sigma) used at a dilution of 1:20,000. The secondary antibody was mouse anti-rabbit IgG-HRP polyclonal antibody (sc-2357; Santa Cruz Biotechnology) used at a dilution of 1:10,000.

### Statistical analysis

All results represent one of at least three independent experiments with similar outcomes. All data are expressed as the mean ± standard error of mean. One-way analysis of variance (ANOVA) and Tukey post hoc test were used to compare groups of data. P≤0.05 was considered to indicate a statistically significant result. GraphPad Software, Version 4.03 (San Diego, CA, USA) was used for the statistical analysis.

## Results

### Effects of TNFα induction with or without R1881 treatment on the expression of p50 and p105 in LNCaP cells

**Figure 1:**
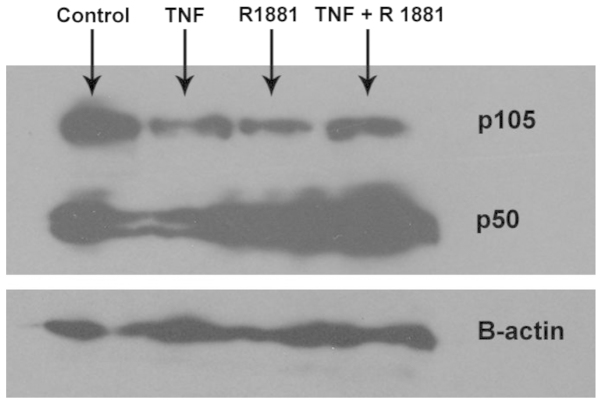
Protein expression of p50 and p105 in LNCaP cells as revealed by western blotting. LNCaP cells were induced with TNFα in the presence or absence of synthetic androgen R1881.

LNCaP cells were treated with TNFα in the presence or absence of R1881, which is a synthetic androgen analog. TNFα induction, R1881 treatment and TNFα induction plus R1881 treatment led to reductions in p105 expression levels. Treatment with TNFα alone caused a slight reduction in the p50 expression level, whereas R1881 treatment increased the protein expression level of p50 in the presence or absence of TNFα (Fig. 1).

### Effects of STAMP1 and STAMP2 transfections with or without TNFα induction on the expression of p50 in DU145 cells

**Figure 2:**
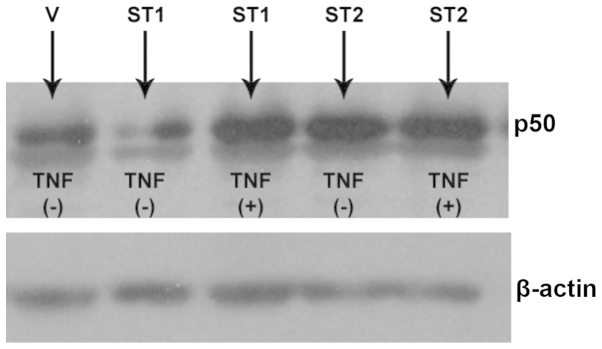
Protein expression of p50 in DU145 cells as revealed by western blotting. DU145 cells were transfected with HisMax-vector (V), HisMax-STAMP1 (ST1) or HisMax-STAMP2 (ST2). Transfected cells were induced by TNFα or not induced.

DU145 cells were transfected with HisMax-vector, HisMax-STAMP1 or HisMax-STAMP2. The transfected cells were then either induced by TNFα or were not induced. HisMAX-STAMP1 transfection decreased the expression level of p50. However, TNFα induction following HisMAX-STAMP1 transfection led to an increase in the expression level of p50. By contrast, HisMAX-STAMP2 transfection increased the expression level of p50, and TNFα induction had no effect on the expression level of p50 in cells transfected with HisMAX-STAMP2 (Fig. 2).

### Effects of TNFα induction on apoptosis-related gene expression in LNCaP cells

**Figure 3:**
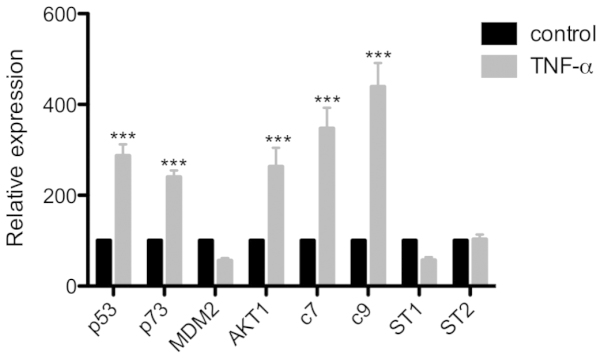
Effects of TNFα induction on the mRNA expression of apoptosis-related genes in LNCaP cells. ^***^P≤0.001 vs. control, compared by one-way analysis of variance followed by Tukey post hoc test.

RT-qPCR amplifications were performed with a panel of apoptosis-related primers following the induction of LNCaP cells with TNFα. Induction with TNFα led to increases in the mRNA levels of the apoptosis-related genes p53, p73, caspase 7 and caspase 9, and the survival-related gene AKT1. Conversely, TNFα induction tended to decrease the mRNA levels of MDM2 and STAMP1; however, the reductions were not significant. The mRNA levels of STAMP2 were unaffected by TNFα induction (Fig. 3).

### Effect of NFκB gene silencing with or without TNFα induction on apoptosis-related gene expression in LNCaP cells

Cells were transfected with siNFκB construct or scrambled control for 1 or 4 days. The cells transfected for 1 day were induced by TNFα or were not induced for a further 24 h in serum medium. The cells transfected for 4 days were induced by TNFα or not induced for 24 h at the third day of transfection.

**Figure 4:**
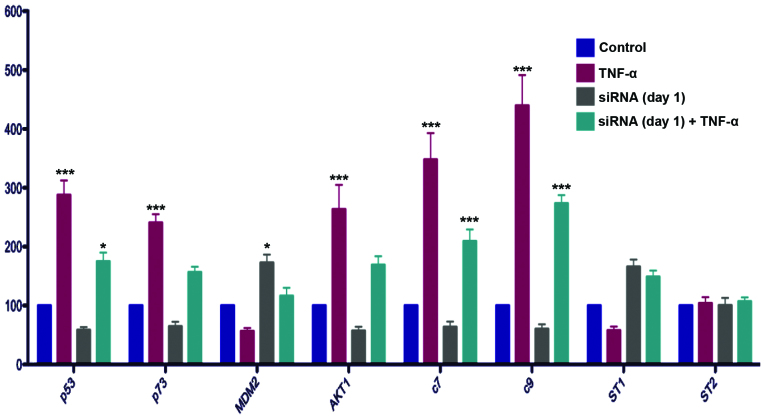
Effect of NFκB gene silencing (day 1) with or without TNFα induction on the expression of apoptosis-related genes in LNCaP cells: Cells were transfected with siNFκB construct or scrambled control for 1 day. Transfected cells were induced by TNFα or were not induced for a further 24 h in serum medium. ^*^P≤0.05, ^***^P≤0.001 vs. control, compared by one-way analysis of variance followed by Tukey post hoc test.

**Figure 5:**
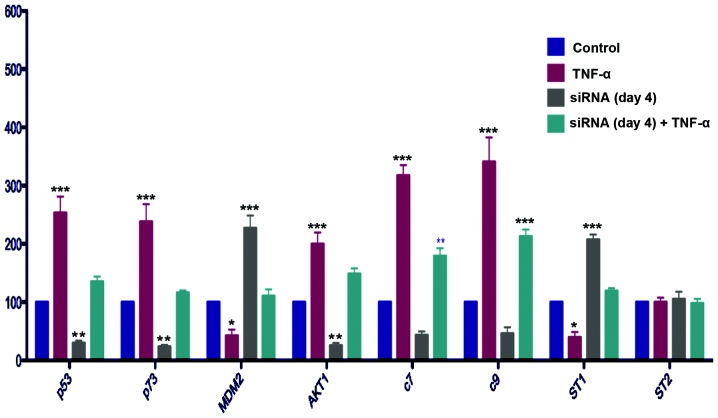
Effect of NFκB gene silencing (day 4) with or without TNFα induction on the expression of apoptosis-related genes in LNCaP cells: Cells were transfected with siNFκB construct or scrambled control for 4 days. Transfected cells were induced by TNFα or were not induced on day 3. ^*^P≤0.05, ^**^P≤0.01, ^***^P≤0.001, compared by one-way analysis of variance followed by Tukey post hoc test.

TNFα induction increased the mRNA levels of p53, p73, AKT1 and caspases 7 and 9, and also tended to decrease the mRNA levels of MDM2 and STAMP1 in the LNCaP cells transfected with scrambled control (Figs. 4 and 5).

Silencing of the NFκB gene decreased the mRNA levels of p53 (Fig. 5). NFκB gene silencing also attenuated the effect of TNFα induction on the mRNA levels of p53 at day 1 (Fig. 4). Silencing of the NFκB gene inhibited the effect of TNFα induction on the mRNA levels of p53 at day 4 (Fig 5).

The effects of NFκB gene silencing on p73 were similar to those on p53. Specifically, NFκB gene silencing decreased the mRNA levels of p73 and these results showed a statistically significant difference between the scrambled control and NFκB gene-silenced groups on day 4 (Fig. 5). In addition, NFκB gene silencing inhibited the effect of NFκB induction on the mRNA levels of p73 (Figs. 4 and 5).

Notably, silencing the NFκB gene decreased the mRNA levels of AKT1, which is known to be a survival gene, at day 4. In addition, it inhibited the effect of TNFα induction on the mRNA levels of AKT1 (Figs. 4 and 5).

Comparison of the MDM2 mRNA levels between the scrambled control and NFκB gene-silenced groups showed that silencing the NFκB gene increased the mRNA levels of MDM2 at both transfection times (Figs. 4 and 5). Silencing the NFκB gene inhibited the effect of TNFα on the mRNA levels of MDM2 on days 1 and 4 (Figs. 4 and 5).

The effects of NFκB gene silencing in the presence or absence of NFκB induction on caspase 7 and 9 were also investigated. Silencing the NFκB gene tended to decrease the mRNA levels of caspase 7 and 9, although the reductions were not statistically significant (Figs. 4 and 5). NFκB silencing decreased the mRNA levels of caspase 7 and 9 in the TNFα-induced cells (Figs. 4 and 5).

NFκB gene silencing increased the mRNA levels of STAMP1 at day 4, and reversed the inhibitory effect of TNFα induction on the mRNA levels of STAMP1 at day 4 (Fig. 5).

Neither silencing the NFκB gene nor TNFα induction had any effect on the mRNA levels of STAMP2 (Figs. 4 and 5).

## Discussion

Since, inflammation and cancer are closely related disorders (9), NFκB is a topic of particular interest to researchers (10). The activation of NFκB is generally achieved by chronic exposure to TNFα (11). The activation results in the altered expression of various genes (12), and also the constant expression of TNF receptors R1 and R2 (data not shown). Altered gene expression has been reported in the following cell lines: DU145, which has constitutive NFκB expression, and LNCaP, which has TNF-inducible NFκB expression (13). NFκB is a heterodimeric or homodimeric complex formed from five different subunits that are known as: RelA (p65), Rel B, c-Rel, NFκB1 (p50) and NFκB2 (p52). p50 and p52 subunits are derivatives of large precursor units p105 and p100, respectively. The classical NFκB heterodimer consists of p65 and p50 (14). In the present study we focused on p105 and one of its subunit, p50 (NFκB1). TNFα induction decreased p105 and p50 expression. This result is consistent with previous studies. The LNCaP cell line has androgen receptor expression and is therefore responsive to R1881, an androgen analog. R1881 treatment decreased p105 expression, whereas it increased p50 expression. The DU145 cell line does not have an androgen receptor and ST1 transfection decreased p50 expression. However, TNFα diminished the effect of STAMP1 transfection and STAMP2 transfection increased p50 expression. Furthermore, TNFα induction has no additional effect on p50 in ST2 transfected cells. These results may indicate that further studies may reveal the correlation between androgen stimulation and the survival gene NFκB in prostate cancer. Additionally, STAMP1 and STAMP2 genes may have different and opposite roles on NFκB signaling.

In order to investigate the potential interactions, RT-qPCR amplifications using a panel of primers specific for intrinsic apoptosis were conducted in the present study. Following induction with TNFα, the mRNA levels of p53, which is a tumor suppressor (15), and p73, which is both a suppressor and supporter of cell growth (16), were found to increase. TNFα induction also increased mRNA levels of AKT1, a survival gene. Expression level changes of AKT1 have been previously revealed in prostate cancer cell lines (17). By contrast, the mRNA levels of MDM2, a ubiquitin ligase for p53 (18) and of STAMP1, identified as a p53 negative regulator (unpublished data), were reduced. Caspases 7 and 9 each have distinct roles during intrinsic apoptosis (19,20), and it was observed in the present study that the mRNA levels of caspase 7 and 9 were increased by treatment with TNFα. Silencing of NFκB almost completely inhibited the effects of TNFα induction on the expression of apoptosis related genes. This result implied that NFκB may play an important role on the regulation of apoptosis-related genes in prostate cancer. The activation of NFκB may cause chemoresistance in chemotherapy regimens (21); therefore, alternative reagents for inhibiting NFκB have been investigated (22,23). Besides, the mRNA expression of STAMP1 was also decreased by TNFα induction. To the best of our knowledge, the present study is the first to reveal effect of TNFα induction on STAMP1. Interestingly, NFκB gene silencing increased STAMP1 expression. Regulation of STAMP1 gene expression may be related to the NFκB pathway. Conversely, STAMP2 amplification was not changed by either TNFα induction or NFκB silencing.

The androgen receptor (AR) is a member of the steroid receptor superfamily and a transcription factor. The response elements of prostate specific antigen (PSA) and NFκB are located at the AR promoter region (24), and suggest that NFκB may effect AR expression. The activation of AKT and NFκB is reported to be involved in the progression of prostate cancer from androgen dependence to independence (25,26). These findings, in combination with previous observations (27) indicate that the effective inhibition of NFκB may be critical in providing a targeted pathway for the prevention of prostate cancer.

## Figures and Tables

**Table I:** Genes and primers used as an apoptosis panel for quantitative polymerase chain reaction (qPCR) analysis.

GenBank/Symbol	Description	Gene name	Primer sequence
NM_005163/AKT1	V-akt murine thymoma viral oncogene homolog 1	PKB/PRKBA	Forward: TCCCCCTCAGATGATCTCTCCA Reverse: CGGAAAGGTTAAGCGTCGAAAA
NM_001227/CASP7	Caspase 7, apoptosis- related cysteine peptidase	CMH-1/ICE-LAP3	Forward: AAGTGAGGAAGAGTTTATGGCAAA Reverse: CCATCTTGAAAACAAAGTGCCAAA
NM_001229/CASP9	Caspase 9, apoptosis- related cysteine peptidase	APAF-3/APAF3	Forward: TCCTGAGTGGTGCCAAACAAAA Reverse: AGTGGTTGTCAGGCGAGGAAAG
NM_005427/TP73	Tumor protein p73	P73	Forward: AGCAGCCCATCAAGGAGGAGTT Reverse: TCCTGAGGCAGTTTTGGACACA
NM_000546/TP53	Tumor protein p53 (Li-Fraumeni syndrome)	CYS51STOP/P53	Forward: AGATGGGGTCTCACAGTGTTGC Reverse: ATGTTGACCCTTCCAGCTCCAC
NM_002392/MDM2	MDM2 proto-oncogene, E3 ubiquitin ligase	HDMX/MGC71221	Forward: GGGTTCGCACCATTCTCCTG Reverse: GGCAGATGACTGTAGGCCAAGC
NM_152999.3/STAMP1	STEAP family member 2, metalloreductase (STEAP2), transcript variant 1	STEAP2/STAMP1	Forward: ATAGGAAGTGGGGATTTTGC Reverse: AGATGTCTCAGGTCCCACAA
NM_024636.3/STAMP2	STEAP family member 4 (STEAP4), transcript variant 1	STEAP4/STAMP2	Forward: GCACTTACACTGCTTGC Reverse: CAGTGGTCAAGCCAGTC
NM_002046/GAPDH	Glyceraldehyde-3-phosphate dehydrogenase	G3PD, GAPD	Forward: CATTGCCCTCAACGACCACTTT Reverse: GGTGGTCCAGGGGTCTTACTCC

GenBank accession numbers for reference mRNA sequences, gene names and and descriptions are as provided by the RefSeq database of the National Center for Biotechnology Information.

## Abbreviations

MDM2: E3 ubiquitin ligase
TNF: tumor necrosis factor
NFκB: nuclear factor κB
STEAP: six transmembrane epithelial antigen of prostate
STAMP: six transmembrane protein of prostate
